# Language Mediated Concept Activation in Bilingual Memory Facilitates Cognitive Flexibility

**DOI:** 10.3389/fpsyg.2017.01067

**Published:** 2017-06-28

**Authors:** Anatoliy V. Kharkhurin

**Affiliations:** Department of International Studies, American University of SharjahSharjah, United Arab Emirates

**Keywords:** bilingualism, bilingual memory, creativity, divergent thinking, cognitive flexibility, priming

## Abstract

This is the first attempt of empirical investigation of language mediated concept activation (LMCA) in bilingual memory as a cognitive mechanism facilitating divergent thinking. Russian–English bilingual and Russian monolingual college students were tested on a battery of tests including among others Abbreviated Torrance Tests for Adults assessing divergent thinking traits and translingual priming (TLP) test assessing the LMCA. The latter was designed as a lexical decision priming test, in which a prime and a target were not related in Russian (language of testing), but were related through their translation equivalents in English (spoken only by bilinguals). Bilinguals outperformed their monolingual counterparts on divergent thinking trait of cognitive flexibility, and bilinguals’ performance on this trait could be explained by their TLP effect. Age of second language acquisition and proficiency in this language were found to relate to the TLP effect, and therefore were proposed to influence the directionality and strength of connections in bilingual memory.

## Introduction

The theme of bilingual and multilingual creative cognition receives growing attention in scientific research (Kharkhurin, 2015c). Increasing number of studies look into the cognitive underpinnings of the relationship between bilingualism and creativity. The findings provide support for bilingual advantages in performance on a variety of creativity tasks (review in Kharkhurin, 2012a).

The positive effect of bilingualism on creativity was explained by the creative cognition paradigm (Finke et al., 1992). The conceptual framework of creative cognition rests on the assumption that “ideas and tangible products that are novel and useful are assumed to emerge from the application of ordinary, fundamental cognitive processes to existing knowledge structures” (Ward, 2007, p. 28). In this framework, creative performance can be perceived as a result of application of specific cognitive processes to specific cognitive structures (Ward et al., 1997). Therefore, creative capacity is assumed as an essential property of normative human cognition (Ward et al., 1999). Increase in general cognitive functioning may reinforce an individual’s creative capacities. At the same time, research in bilingualism supplies growing evidence that bilingual development establishes specific structures of the mind that could promote cognitive advantages (review in Nicoladis, 2008). Hence, “if bilingualism <…> results in more elaborate cognitive structures and/or functioning, it may also facilitate creative functioning” (Kharkhurin, 2015a, p. 39). That is, an individual’s acquisition and use of multiple languages may have impact on one’s cognition and therefore facilitate creative cognition (Kharkhurin, 2015c).

A number of factors in bilinguals’ cross-linguistic and cross-cultural experiences were identified as encouraging for the cognitive mechanisms underlying their creative performance. The role of inhibition and facilitation mechanisms of selective attention in bilingual creative cognition was already empirically investigated (Kharkhurin, 2011). The present work aims to investigate another cognitive mechanism—language mediated concept activation (LMCA)—which on the one hand, presumably benefits from a specific architecture of bilingual memory, and on the other, facilitates bilinguals’ divergent thinking.

### Divergent Thinking

Psychometric tradition perceives creative thinking as an ability to start multiple cycles of divergent and convergent thinking (Guilford, 1967). The combined work of these two types of thinking initiates a process that sanctions generation of ideas fulfilling the defining characteristics of a creative product: novelty (i.e., unexpected or original) and utility (i.e., useful or meeting task constraints) (see Sternberg, 1999, for an overview ^1^). Many studies in the field supplied evidence that divergent thinking tests predict certain aspects of creative problem-solving and real world creative achievements. Although, “divergent thinking is not synonymous with creative thinking” (Runco, 1991, p. ix), many studies perceive divergent thinking as a defining component of creative process (Lubart, 2000). Guilford identified four traits of divergent thinking. Flexibility addresses the ability to consider a variety of approaches to a problem simultaneously. Fluency refers to a quantity of ideas or solutions produced to solve a problem. Originality refers to the tendency to produce novel ideas. Elaboration is perceived as the capacity to embellish an idea with details.

The functioning of divergent thinking can be explained by an automatic spreading activation mechanism that simultaneously activates a large number of mental representations. These representations are stored in conceptual memory. The latter is assumed as a pattern of spreading activation (McClelland and Rumelhart, 1985) over a large set of mutually linked units of meaning (or conceptual features) organized in conceptual networks (Lamb, 1999). In this view, mental representations are seen as an emergent property of neural activity in the conceptual system (Bunge, 1980). Any sensory experience as well as any product of our thought process is stored as a pattern of neural activity and leaves a trace in our memory. The spreading activation mechanism transfers activation between conceptual features, providing facilitation for related concepts and inhibition for unrelated ones. This property of the conceptual system was illustrated in priming studies (e.g., Meyer and Schvaneveldt, 1971) that show that semantically related words tend to influence each other. The activation of the conceptual features is assumed an unconscious process. The activation propagates across the conceptual network. Only those features that receive enough activation are selected for conscious processing. The associations between distant mental representations become possible due to the distributed nature of the conceptual system (see Kharkhurin, 2012a, for details).

In light of this discussion, divergent thinking takes place when a large number of conceptual representations are accessed simultaneously. Spreading activation among distributed conceptual representations may establish associations that link often-unrelated ideas represented by concepts from distant categories. Simultaneous activation of these ideas may establish a rich plane of thought from which original and novel ideas might be extracted (Mumford et al., 1991). Thus, divergent thinking refers to the ability to access a large number of unrelated conceptual representations from distant conceptual categories simultaneously.

### How the Structure of Bilingual Memory Facilitates Divergent Thinking

There is empirical evidence that bilinguals outperform their monolingual counterparts on divergent thinking tests (review in Ricciardelli, 1992b; Kharkhurin, 2012a). This effect was found for all four traits mentioned above: flexibility (e.g., Carringer, 1974; Konaka, 1997; Kharkhurin, 2008; Adi-Japha et al., 2010; Cushen and Wiley, 2011), fluency (e.g., Jacobs and Pierce, 1966; Carringer, 1974; Ricciardelli, 1992a; Kharkhurin, 2008; Kostandyan and Ledovaya, 2013), originality (e.g., Cummins and Gulutsan, 1974; Okoh, 1980; Konaka, 1997; Kharkhurin, 2009), and elaboration (e.g., Torrance et al., 1970; Srivastava and Khatoon, 1980; Kharkhurin, 2008; Lee and Kim, 2011).

The present work argues that the specific architecture of bilingual memory facilitates greater spreading activation between conceptual representations and thereby stimulates divergent thinking. This may be accomplished through LMCA (Kharkhurin, 2007, 2008, 2012a) that postulates that unrelated conceptual representations are activated through bilinguals’ two languages. This section presents a model of bilingual memory that allows the LMCA and thereby facilitates spreading activation between unrelated concepts.

#### Distributed Lexical/Conceptual Feature Model of Bilingual Memory

**FIGURE 1:**
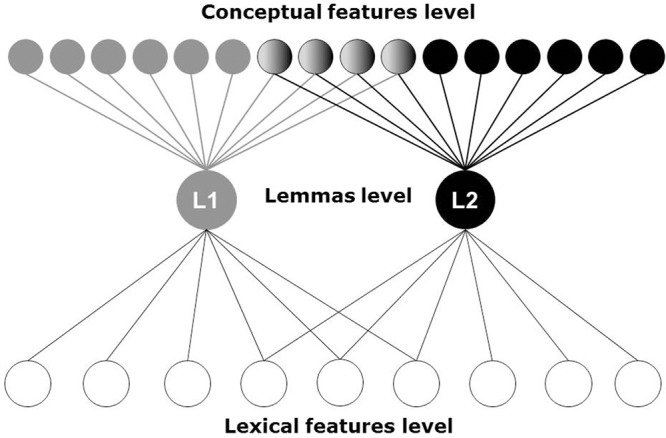
The distributed lexical/conceptual feature model of bilingual memory consists of three levels of representation: a conceptual feature, a lexical feature, and a lexical-semantic (lemma). L1, first language; L2, second language. Adopted from Kroll and de Groot (1997).

This study assumes a structure of bilingual memory as presented in distributed lexical/conceptual feature model (see Kroll and de Groot, 1997, for a detailed description). This model (see **Figure 1**) presents bilingual memory as a dynamic system with three levels of representation. A *conceptual features* level contains representations of meaning. A *lexical features* level contains only aspects of word form (e.g., phonetic, orthographic), but not the word meanings. A *lexical-semantic* (*lemmas*) level relates the word forms and their meaning. Both conceptual and lexical features levels are language non-specific (i.e., shared across both languages), and the lemmas level is specific for each language.

The conceptual features level includes distributed conceptual representations. The lexical features level includes distributed lexical representations. The distributed nature of the lexical features supposes that words in both languages can share the same word forms. For example, a word “marker” in English shares phonetic features with a Russian word “marka” /stamp/ (Spivey and Marian, 1999). The lexical-semantic level includes language specific lemmas. Lemma can be seen as an abstract lexical unit that mediates conceptual representation and word form. In language production, lemma is selected after the word has been retrieved mentally, but before any information about the word form is accessed. The lemma contains some information about the meaning and syntactic role of a word, but not about its form (Kroll and de Groot, 1997).

The spreading activation discussed above is assumed as a mechanism of communication between all three levels. On the one hand, this mechanism supports the distributed feature representation at each level, and on the other hand, it encourages parallel processing and communication between the features at different levels. The cross-language communication is established at the lexical feature level due to assumption that the word forms for both languages are shared. Activation of common word forms results in activation of lemmas from both languages. The connection from both languages to the concepts is established through direct links from L1 and L2 lemmas to the conceptual system.

#### Language Mediated Concept Activation

The proposed structure of bilingual memory is assumed as foundation for the LMCA. Kharkhurin (2008) described the idea of the LMCA as “based on the assumption that translation equivalents automatically activate each other through shared conceptual representations (e.g., concept mediated translation in Kroll and de Groot, 1997). Although translation equivalents share most of the conceptual features, these representations are not identical (e.g., Paradis, 1997). Variations in the conceptual representations of translation equivalents may result in the simultaneous activation of additional concepts, which eventually may produce a large pattern of activation over unrelated concepts from different categories” (p. 235). The activation of these concepts is assumed to take place through two lexical levels (therefore, *language* mediated concept activation): lexical-semantic (lemma) and lexical features levels.

**FIGURE 2:**
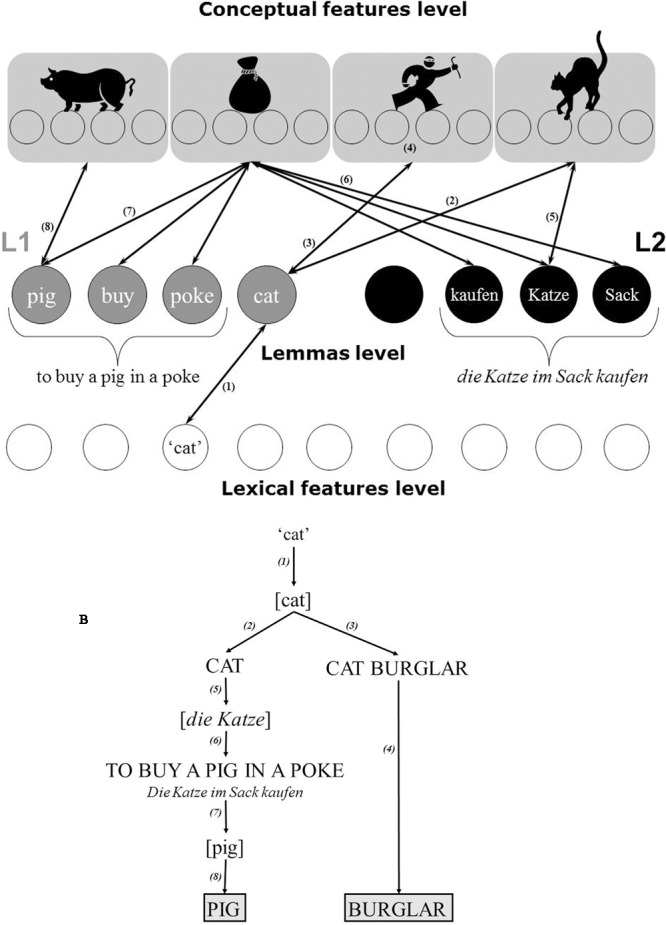
Illustration of a lemma mediated spreading activation underlying the language mediated concept activation. Schematic representation of **(A)** a fragment of bilingual memory structure, and **(B)** the information flow in bilingual memory. The bilingual memory consists of the distributed lexical features level, language specific lemma level, and distributed conceptual features level. The presentation of the English word “cat” activates a lemma [cat] in English lexicon (1). This lemma in turn activates conceptual features that represent the literal meaning of a cat (2) as well as the conceptual representation of the additional meaning of the lemma [cat] such as the one in the “cat burglar” (3). Thereby the conceptual representation of BURGLAR is activated (4). At the same time, the conceptual representation of a cat sends partial activation back to the lemma level in German lexicon thereby activating a lemma [*die Katze*] (5). This lemma sends partial activation to the conceptual representation of the additional meaning of the lemma [*die Katze*] such as the one in “*die Katze im Sack kaufen*” (6). Accordingly, the latter sends partial activation back to the lemma level in English lexicon thereby activating a set of lemmas corresponding to the idiom “to buy a pig in a poke.” Therefore, among the others, a lemma [pig] is activated (7) and in turn, triggers its corresponding conceptual features (8). Thus, the LMCA results in a simultaneous activation of two unrelated conceptual representations of a pig and a burglar. Adopted from Kharkhurin (2007).

First, “conceptual representations sharing the same lemmas can be activated” (Kharkhurin, 2007, p. 198). Kharkhurin proposed the following workflow for the lemma mediated concept activation: “A word in L1 activates corresponding lemmas in the L1 lexicon, which in turn, activate the corresponding conceptual features. The conceptual features send partial activation back to the L2 lexicon, which activates the corresponding L2 lemmas. These lemmas, once activated, may send partial activation to the conceptual features representing concepts that share this lemma with the target word” (p. 198). He illustrated this with an example: “the presentation of an English word “cat” to German–English bilingual activates a lemma [cat] in the English lexicon (see **Figure 2A**). This lemma in turn sends activation to conceptual features that represent the literal meaning of a CAT; additionally, it may send a partial activation to the conceptual representation of the alternative meaning of the lemma [cat] such as the one in the “cat burglar.” Thus, the conceptual representation of a BURGLAR is activated. At the same time, the conceptual representation of a CAT sends partial activation back to the lemma level in the German lexicon thereby activating the lemma [*die Katze*] <(German word for a cat)>. This lemma, once activated, may in turn send partial activation to the conceptual representation of the additional meaning of the lemma [*die Katze*] such as the one in “*die Katze im Sack kaufen*.” Accordingly, the latter may send partial activation back to the lemma level in the English lexicon thereby activating a set of lemmas corresponding to the idiom “to buy a pig in a poke,” an English translation equivalent to the German expression. Therefore, among the others, a lemma [pig] is activated, which in turn triggers its corresponding conceptual features. As a result, a large pattern of conceptual representations is activated that allows simultaneous exploration of unrelated concepts (such as BURGLAR and PIG) from distant categories (such as [crime] and [animal])” (p. 198–199). **Figure 2B** presents an activation flow between lemmas and conceptual features level.

Second, “conceptual representations sharing the same word forms can be activated” (Kharkhurin, 2007, p. 198). Kharkhurin proposed the following workflow for the lexical features mediated concept activation:

“Words that share the same word forms (e.g., orthographic, phonological) may activate each other in the same way as the words with similar lexical properties activate each other in the monolingual memory (e.g., Allopenna et al., 1998). This assumption was inspired by the findings of eye-tracking studies showing that cross-linguistic homophones tend to activate each other (e.g., Marian and Spivey, 2003). Marian and her colleagues recorded the eye movements of Russian–English bilinguals while giving them instructions in target language (e.g., “*Podnimi marku*” /Pick up the stamp/). The recording showed that while participants’ eyes focused on the stamp they also looked briefly at the objects with a phonologically similar name in non-target language (e.g., a marker, /*flomaster*/ in Russian). Similar results were obtained in research on cross-linguistic orthographic priming with French–English bilinguals (Bijeljac-Babic et al., 1997). In the lexical decision task, orthographically related words in French and English tended to inhibit each other, indicating that printed strings of letters can simultaneously activate lexical representations in each of the bilinguals’ languages. Thus, semantically unrelated words in bilingual lexicons can activate each other if they share similar lexical features. A set of distributed lexical features shared by both lexicons can send the activation to the lemmas in different languages thereby initiating the lemma mediated concept activation. For example, the oral presentation of the English word “marker” to Russian–English bilinguals may activate a set of phonological features that are present in both “marker” and “*marka*.” These features therefore activate the lemma [marker] in the English lexicon and the lemma [stamp] in the Russian lexicon. These lemmas in turn activate the conceptual representations of the marker and the stamp, which appear to be unrelated in a monolingual lexicon” (p. 200).

Thus, the LMCA implies that activation flow in the conceptual system mediated by the linguistic systems establishes the links between various, often unrelated, concepts. In this manner, conceptual representations from distant categories can be activated simultaneously. This, in turn, may enhance divergent thinking capacities in bilingual individuals.

### Present Study

The algorithms proposed for lemma and lexical features mediated concept activation are rather speculative and require empirical investigation. The purpose of the present study is to test the lemma mediated concept activation algorithm empirically.

#### Translingual Priming as an Operational Definition of the LMCA

**FIGURE 3:**
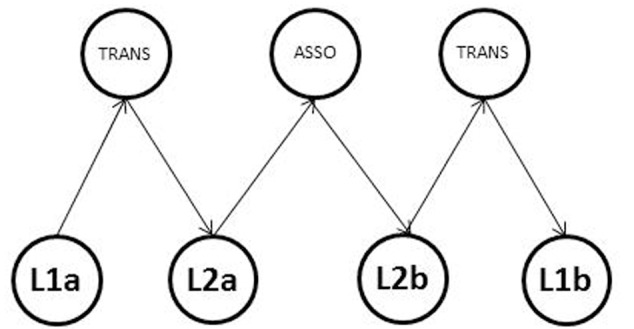
A presumed functioning of the language mediated concept activation. TRANS units represent the conceptual representations shared by translation equivalents. ASSO unit represents conceptual representations shared by associative words. L2a unit represents a translation equivalent of L1a unit, L2b unit represents an associate of L2a unit, and L1b unit represents a translation equivalent of L2b unit.

We operationalize this algorithm as translingual priming (TLP), which involves three steps (see **Figure 3**). First, a target word in L1 activates its conceptual representation, which in turn activates its translation equivalent in L2. The theoretical considerations underlying this step are based on concept mediation model (Potter et al., 1984). The model assumes that words in both languages are directly connected to the underlying concepts and that these language systems are linked through the shared conceptual representations. The cross-language studies using translation priming paradigms (see Altarriba and Basnight-Brown, 2007, for a review) support this assumption by demonstrating that automatic spreading activation takes place between translation equivalents. **Figure 3** presents this connection as L1a–TRANS–L2a, where TRANS presents shared conceptual representation of L1 and L2 translation equivalents.

Second, the translation equivalent in L2 activates its conceptual representation and subsequently, due to distributed nature of this representation, activates its semantically related associate in the same L2 (L2a–ASSO–L2b connection in **Figure 3**). The within-language priming studies (e.g., Meyer and Schvaneveldt, 1971) support this assumption by demonstrating that semantically related words prime each other. As a result, we expect an activation flow between a target word in L1 and its semantically related word in L2. The cross-language studies using semantic priming paradigms (see Kroll and Tokowicz, 2005, for a review) support this expectation by demonstrating that automatic spreading activation takes place between semantically related words in different languages.

Finally, the associate in L2 activates its conceptual representation, which in turn activates its translation equivalent in L1 (similar to the first step; L2b–TRANS–L1b connection in **Figure 3**).

Thus, the activation spreads between semantically unrelated words in L1, which appear to relate through L2. In this case, the TLP effect occurs between two words in the same language, which are not semantically related in this language, but related through another bilingual’s language. The evidence of the activation flow between semantically unrelated words within the same language would signify the LMCA.

#### The Effect of the LMCA on Divergent Thinking Traits

As discussed above, the LMCA in bilingual memory is conceived as facilitating divergent thinking. Two divergent thinking traits appear to benefit from this mechanism: flexibility and fluency. Recall that flexibility addresses the ability to consider simultaneously a variety of approaches to a problem, and fluency taps into the capacity to produce a large quantity of ideas or solutions to a problem. The LMCA triggers simultaneous activation of conceptual representations from distant categories. Simultaneous activation of multiple conceptual planes presumably allows an individual to consider various approaches to a problem; that is, it promotes flexibility. Concurrently, simultaneous activation of unrelated concepts presumably allows an individual to produce a large amount of solutions to a problem; that is, it promotes fluency.

Based on these assumptions, three hypotheses were advanced in this study. First, in line with the previous research in bilingual creative cognition, we predict that bilinguals outperform monolinguals on flexibility and fluency components of divergent thinking test. Second, we hypothesize that TLP effect predicts bilinguals’ performance on these components. Third, we hypothesize that due to the LMCA bilinguals demonstrate greater TLP effect than monolinguals.

Finally, research with bilinguals at different stages of L2 acquisition (Potter et al., 1984; Kroll and Curley, 1988; Chen and Leung, 1989) demonstrated that at the earlier stages of language learning there is more reliance on translation equivalents between L1 and L2, whereas at the later stages direct concept mediation is possible. This transition depends on the linguistic skills in L2. To account for these findings, Kroll and Stewart (1994) proposed an asymmetry in lexical access. During early stages of L2 acquisition, the connection from L1 to the concepts is stronger than the one from L2, and learners rely on L2–L1 connection to access meanings in L2. As learners become more proficient, the connection between L2 and conceptual system intensifies so that they rely more on this connection rather than on the one mediated by L1. The reinforced L2–concept connection weakens L1–L2 connection. That is, experience with L2 reduces the asymmetry in lexical access, In terms of the LMCA model, greater proficiency in L2 may strengthen the L2 lemma–concept connections. Since the TLP relies on direct lexical access in both languages (i.e., L1–concept–L2), it should benefit from greater proficiency in L2. Hence, the forth hypothesis predicts that the TLP effect is modulated by L2 proficiency.

## Materials and Methods

### Ethic Statement

This study was carried out in accordance with the recommendations of the AUS IRB guidelines with written informed consent from all subjects. All subjects gave written informed consent in accordance with the Declaration of Helsinki. The protocol was approved by the AUS IRB Committee.

### Participants

The bilingual sample comprised of 58 Cherepovets State University (Russia) bilingual students (4 male and 54 female) aged between 17 and 25 (*M* = 19.69, *SD* = 1.61) who were recruited through posters that advertised the need for Russian–English bilinguals. The monolingual sample comprised of 28 individuals from the same pool (5 male and 23 female) aged between 20 and 25 (*M* = 21.43, *SD* = 1.26). Considering unequal distribution of male and female participants in the samples, they were compared on all measures used in the study. No significant difference was found between male and female groups on these measures. The bilingual sample was significantly younger than the monolingual sample (Δ*M* = -1.74, *t* = -5.01, *p* < 0.001).

All participants were native speakers of Russian. However, bilinguals scored significantly higher than their monolingual peers did on the language proficiency test of Russian (see description of the test below; *M* = 99.57, *SD* = 9.54 for bilingual and *M* = 94.36, *SD* = 13.83 for monolingual group, *t* = 2.04, *p* < 0.05). Bilingual participants reported that they started learning English at school at the age of 9.46 (*SD* = 3.66) years old on average, and mastered it for 10.23 (*SD* = 4.00) years on average.

The significant differences between bilingual and monolingual samples in age and proficiency in Russian were taken into account in further analyses.

### Procedure

The experiment comprises of four sessions. During the first session, participants completed a biographical questionnaire, language proficiency assessment, divergent thinking assessment, and fluid intelligence assessment. To prevent the fatigue effect, the order of presenting these tests was counterbalanced. During the following three sessions, participants were tested on the TLP test. The test consists of three parts, each of which uses the same target stimuli (see Translingual Priming Test). To prevent an uncontrolled priming effect, these parts were presented in a counterbalanced order with a minimum of 2-week lag.

### Instruments

#### Biographical Questionnaire

Multilingual and Multicultural Experience Questionnaire^2^ (Kharkhurin, 2012a) has been translated by the author into Russian and administered to determine participants’ linguistic and cultural background. They received a paper version of the questionnaire that, among other questions, obtained data on languages spoken by participants, their assessment of linguistic skills in each of these languages, and age of acquisition and length of usage of these languages.

#### Language Proficiency Assessment

Participants’ proficiency in Russian and/or in English was evaluated by a test of productive vocabulary, the internet-based Picture Naming Test (iPNT; Kharkhurin, 2012b; http://harhur.com/ipnt/). This test was used in most of the author’s studies. Kharkhurin and Altarriba (2016) described this test as following:

“Language proficiency was assessed by the accuracy of the participants’ written responses to 120 pictures of simple objects, a technique similar to the Boston Naming Test (Kaplan et al., 1983) and that used by Lemmon and Goggin (1989). This technique was shown to be a reliable measure of language proficiency that highly correlated with participants’ self-rating of linguistic skills and their self-assessment of degree of bilingualism (Kharkhurin, 2008). Each response was scored either 1 or 0, so that the maximum number of points for picture naming was 120. Participants had eight minutes to complete this test” (p. 1083).

#### Divergent Thinking Assessment

The divergent thinking assessment was used in most of the author’s studies. Kharkhurin and Altarriba (2016) described it as following:

“Participants’ creative abilities were assessed using the Abbreviated Torrance Test for Adults (ATTA; Goff and Torrance, 2002). The ATTA was developed on the basis of the Torrance Tests of Creative Thinking (Torrance, 1966). It consists of activities utilizing the same rationale as activities in the original test, but in abbreviated form and requires considerably less testing time, which is particularly beneficial when administering it to adults. The ATTA was employed in a series of studies of bilingual creativity (Kharkhurin, 2008, 2009, 2010a,b, 2011; Kharkhurin and Wei, 2014) and demonstrated good assessment of bilinguals’ creative behavior. 0 The standard ATTA has three 3-minute paper and pencil activities preceded by a written instruction that explains general guidelines and encourages participants to use their imagination and thinking abilities. In Activity 1, participants were asked to suppose that they could walk on air or fly, and then to identify the troubles that they might encounter. This activity provided verbal fluency and originality scores. In Activity 2, participants were presented with two incomplete figures and were asked to draw pictures with these figures and to attempt to make these pictures as unusual as possible. This activity provided nonverbal fluency, originality, and elaboration scores. In Activity 3, the participants were presented with a group of nine triangles arranged in a 3×3 matrix and were asked to draw as many pictures or objects as they could using those triangles. This activity provided nonverbal fluency, originality, elaboration, and flexibility scores.

The standard ATTA assessment consists of four divergent thinking traits: fluency, originality, elaboration, and flexibility. Fluency measures the ability to produce quantities of ideas, which are relevant to the task instructions. The fluency score in Activity 1 provided a verbal fluency score; a sum of fluency scores in Activities 2 and 3 provided a nonverbal fluency score. Originality measures the ability to produce uncommon ideas, or ideas that are totally new or unique. The originality score in Activity 1 provided a verbal originality score; a sum of originality scores in Activities 2 and 3 provided a nonverbal originality score. Elaboration measures the ability to embellish ideas with details. The sum of elaboration scores in Activities 2 and 3 provided a nonverbal elaboration score. Finally, flexibility measures the ability to process information or objects in different ways, given the same stimulus. A nonverbal flexibility score was obtained from Activity 3.

In addition, the scores for five verbal criterion-referenced creativity indicators were obtained from responses in Activity 1 (richness and colorfulness of imagery, expressions of feelings and emotions, future orientation, humor/conceptual incongruity, and provocative questions), and ten nonverbal criterion-referenced creativity indicators were obtained from responses in Activities 2 and 3 (openness/resistance to premature closure, unusual visualization, movement and/or sound, richness and colorfulness of imagery, abstractness of title, environment for objects/articulateness in telling story, combination/synthesis of two or more figures, internal visual perspective, expressions of feelings and emotions, and fantasy). Each of these 15 scores was 0 if the indicator does not occur, 1 if the indicator appears once, or 2 if the indicator appears more than once.

Two independent raters assessed participants’ divergent thinking abilities using the standard ATTA assessment procedure (Goff and Torrance, 2002). To ensure that both raters used the same rationale, the following procedure was applied. A creativity index was computed as a sum of four norm-referenced and two criterion-referenced creativity indicators scores. The index represented a composite measure of overall creativity” (pp. 1084–1085).

The correlation between the composite creativity indexes produced by both raters was significantly high (*r* = 0.85, *p* < 0.001). This indicated that the raters used the same rationale, and their ratings were comparable. Note that the composite creativity index was used only to determine the inter-rater correlation. In the further analyses, we used the averages of respective norm-referenced scores produced by both raters.

#### Fluid Intelligence Assessment

Participants’ fluid intelligence (Gf) was assessed by the standard Culture Fair Intelligence Test (CFIT; Cattell, 1973). Similar to most of the author’s studies, the present study used Scale 3 Form A of the CFIT. Kharkhurin (2012a) described the test as following:

The scale “contains four subtests involving different perceptual tasks, so that the composite intelligence measure does not rely on a single skill. Each subtest was preceded by the instructions orally presented by the experimenter followed by examples that ensure understanding of the task requirements by the participants. In the Series subtest, participants were presented with incomplete progressive series. Their task was to select, from among the choices provided, à figure, which best continues the series. In the Classification subtest, participants were presented with five figures, among which they had to identify correctly two figures, which were in some way different from the other three. In the Matrices subtest, the task was to complete correctly the design of a matrix presented at the left of each row. The final subtest, Conditions required participants to select from the five choices provided the one which duplicated the conditions given in the far left box.

The raw scores obtained from all four subtests were summarized and subsequently transformed into a normalized Gf score by the recommended procedure (Cattell, 1973), which took age-related norms into account. The CFIT manual reports the reliability coefficients for consistency over items as 0.74, and test–retest consistency as 0.69. It also reports the concept validity as 0.85, and concrete validity calculated as a direct correlation with other tests of intelligence as 0.66. In addition, the manual reports a number of studies demonstrating insignificant effects of sociocultural environment on test scores” (p. 77).

#### Translingual Priming Test

This test employs priming paradigm (Meyer and Schvaneveldt, 1971) with lexical decision task. Five types of prime-target stimulus pairs were used: critical related (CR), critical unrelated (CU), non-word–target (NWT), fillers, and word–non-word (WNW). CR word pairs were composed in such a way that the prime and the target would not be semantically related in Russian, but could be related through their respective English translation equivalents. A set of English pairs was constructed by a native speaker of English (e.g., “march”—“April”) so that the prime in each pair has a double meaning (e.g., to march and March) and the target is an association to the second meaning of the prime (e.g., March). Among these pairs, those that have an association rate greater or equal to 0.01 were selected. The association rate was calculated as a frequency of occurrence of the target word as a reaction to the prime word as a stimulus (according to Edinburgh Word Association Thesaurus, Kiss et al., 1973) divided by the total number of responses to the prime word as a stimulus. Further, the English words were translated into Russian using *ABBYY Lingvo 12* software and translations were verified by two English–Russian speakers. Subsequently, each Russian pair was controlled for the lack of association between the prime and the target using Russian Association Dictionary (Karaulov et al., 2002). The resulting Russian pairs formed the CR word pairs (see **Appendix A**). The prime and the target in these pairs are not semantically related in Russian, but can be related through the respective English translation equivalents. CU word pairs were constructed from words unrelated to the CR target words as a prime, and the CR target words as a target (e.g., 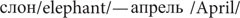). After removing the outliers, the CU primes matched the CR primes in length measured by a number of letters (Δ*M* = -0.13, *t* = -0.40, *p* = 0.69) and frequency obtained from Sharoff’s (2005) Russian Word Frequency Dictionary (Δ*M* = 10.15, *t* = 0.85, *p* = 0.40; see **Table 1**). *Fillers* were constructed using random words (e.g., 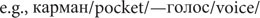). They matched CR and CU pairs in word length and frequency (see **Table 1**). Special caution was taken not to include semantically related pairs (association frequency of these words equals 0.00 as measured by Russian Association Dictionary; Karaulov et al., 2002).

**Table 1:** The mean (with standard deviations in parenthesis) word length and frequency (obtained from Russian Word Frequency Dictionary; Sharoff, 2005) of primes and targets in critical related (CR), critical unrelated (CU), non-word–target (NWT), filler, and word–non-word (WNW) pairs.

Stimulus	Length	Frequency
CR prime	5.38 (1.25)	29.56 (49.20)
CU prime	5.50 (0.89)	19.41 (29.45)
NWT prime	5.67 (1.27)	
CR/CU/NWT target	5.33 (1.40)	82.92 (110.41)
Filler prime	5.38 (0.97)	41.97 (45.78)
Filler target	5.78 (1.32)	57.81 (56.78)
WNW prime	5.31 (1.11)	52.56 (126.91)
WNW target	5.63 (0.91)	

The other two types of stimuli used non-words, which were generated by Russian Nonword Generator developed by the Cherepovets State University’s IT department. All non-words were pseudohomophones five to seven letters in length with orthographically existing onsets and bodies, polymorphemic syllables, and legal bigrams. NWT pairs were created using non-words as primes and the CR target words as targets (e.g., 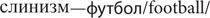). WNW pairs were composed from randomly selected words that match the CR and CU primes in length and frequency (see **Table 1**) as primes and non-words as targets (e.g., 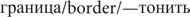).

Total, 48 WNW and 24 of each other type of pairs were constructed. Each participant was tested on each CR, CU, and NWT pairs. Recall that the same words were used as targets in these pairs. To prevent repetition priming, three stimulus lists were constructed so that a target word appears only once on each list. Thus, within each list the target words in CR, CU, and NWT pairs were different from one another. These lists were used in different versions of the test, which were presented to each participant in a counterbalanced order with a minimum 2-week lag. The same fillers and WNW pairs were used in each list. Thus, each version of the test presented a participant with 96 stimulus pairs: 8 CR, 8 CU, 8 NWT, 24 fillers, and 48 WNW. Overall, each word list resulted in a relatedness proportion (i.e., the proportion of related prime–target trials out of all prime–target word trials) of 0.33 and a non-word ratio (the proportion of non-words out of all non-word and unrelated word pairs) of 0.60.

This test used procedure similar to the one in Basnight-Brown and Altarriba (2007). Task instructions appeared before the practice block. The latter consisted of a set of six practice trials that contained word and non-word pairs not included in the experimental lists. The same practice trials were used for all participants. The experimental block started only after participants produced more than 85% of correct responses in the practice block. Participants were given an opportunity to ask additional questions after the practice block. Each trial started with a focusing “+” sign that appeared for 500 ms in the center of the screen. The focusing sign was replaced by the prime, which appeared for 100 ms. The prime was then replaced by the target. Stimuli were displayed in black lowercase letters on a white background. The participants were instructed to press the green key on the response box with their corresponding index finger if the target letter string was a real word and the red key with their other index finger if a non-word appeared. The position (left, right) of the red and green keys was counterbalanced across the participants. The participants were encouraged to respond as accurately and as quickly as possible. The target stayed on the screen until the participants pressed the key or for a maximum of 1,500 ms. In case of incorrect or no response, the word “error” appeared on the screen for 750 ms. The intertrial interval was 2,000 ms.

The entire test took about 10–15 min to complete. It was administered with the E-Prime 2.0 Studio program on 19″ Dell LCD monitor located at 60 cm from a viewer. The responses were recorded by the PST serial response box; only two keys on the response box were used, marked in red and green, respectively.

## Results

### TLP Effect

The mean accuracy rate data from the TLP test are presented in **Table 2**. The repeated measure ANOVA conducted on the accuracy for a stimulus type (CR, CU, NWT, fillers, WNW) as within-subject factor revealed a significant main effect [*F*(4,82) = 25.90, *p* < 0.001, η^2^ = 0.56]. The *post hoc* pairwise comparison revealed significantly greater accuracy on CR than on filler (Δ*M* = 0.06, *SE* = 0.01, *p* < 0.001) and WNW (Δ*M* = 0.03, *SE* = 0.01, *p* < 0.001) pairs; and on CU than on filler (Δ*M* = 0.07, *SE* = 0.01, *p* < 0.001) and WNW (Δ*M* = 0.04, *SE* = 0.01, *p* < 0.001) pairs. This demonstrates that participants made fewer errors on critical pairs (CR and CU) than on control pairs. No significant difference was found in accuracy rates for critical pairs.

**Table 2:** The mean (with standard deviations in parenthesis) accuracy rate and RT data for critical related (CR), critical unrelated (CU), non-word–target (NWT), filler, and word–non-word (WNW) conditions, *N* = 86.

Condition	Accuracy	RT (ms)
CR	0.96 (0.06)	561.10 (60.72)
CU	0.95 (0.05)	558.51 (60.28)
NWT	0.94 (0.14)	570.92 (63.95)
Filler	0.90 (0.07)	611.34 (62.26)
WNW	0.93 (0.07)	657.86 (64.70)

Similar to Basnight-Brown and Altarriba (2007), mean reaction times (RTs) for correct responses were calculated after removing outliers. Response latencies that were beyond 3 SD (under 127.86 ms or over 1,118.37 ms) were removed from the data (1.72%). **Table 2** presents the RT data for the correct responses after the outliers were removed. The repeated measure ANOVA conducted on the RT for a stimulus type (CR, CU, NWT, fillers, WNW) as within-subject factor revealed a significant main effect [*F*(4,82) = 110.54, *p* < 0.001, η^2^ = 0.84]. The *post hoc* pairwise comparison revealed significantly faster RT on CR than on NWT (Δ*M* = -9.83, *SE* = 4.05, *p* < 0.05), filler (Δ*M* = -50.25, *SE* = 2.96, *p* < 0.001), and WNW (Δ*M* = -96.76, *SE* = 5.25, *p* < 0.001) pairs; and on CU than on NWT (Δ*M* = -12.41, *SE* = 3.62, *p* < 0.001), filler (Δ*M* = -52.84, *SE* = 3.11, *p* < 0.001), and WNW (Δ*M* = -99.35, *SE* = 5.36, *p* < 0.001) pairs. This demonstrates that participants responded faster to critical pairs (CR and CU) than to control pairs. No significant RT difference was found between critical pairs.

The RTs for critical pairs were used in the further analysis. The TLP effect score was calculated as the RT difference between responses to CU and CR pairs; greater score signifying greater TLP effect.

### Hypothesis I: Bilinguals Outperform Monolinguals on Flexibility and Fluency

**Table 3** shows age of English acquisition, iPNT scores for Russian and English, TLP effect, ATTA scores, and CFIT scores for bilingual (first column) and monolingual (forth column) groups.

**Table 3:** The mean (with standard deviations in parenthesis) age of English acquisition, iPNT scores for Russian and English, TLP effect, ATTA scores, and CFIT scores for bilingual (overall, high English and moderate English) and monolingual groups.

	Overall bilingual	High English bilingual	Moderate English bilingual	Monolingual
N	58	32	26	28
Age of English acquisition	9.46 (3.66)	7.92 (2.59)	11.35 (3.94)	–
iPNT English	57.50 (19.44)	70.84 (13.44)	41.08 (11.37)	–
iPNT Russian	99.57 (9.54)	99.81 (10.18)	99.27 (8.87)	94.36 (13.83)
TLP effect	–2.77 (24.99)	3.99 (23.93)	–11.08 (24.16)	–2.20 (30.23)
Fluency	16.42 (2.13)	16.44 (2.13)	16.40 (2.17)	16.18 (2.13)
Flexibility	15.44 (1.92)	15.42 (2.01)	15.46 (1.85)	14.41 (1.66)
Elaboration	14.89 (2.26)	15.03 (2.36)	14.71 (2.17)	14.39 (2.34)
Originality	14.99 (2.29)	14.75 (2.14)	15.29 (2.47)	14.54 (2.38)
Gf	108.84 (13.50)	110.91 (14.60)	106.31 (11.78)	105.86 (13.25)

To test the first hypothesis, a series of ANOVAs were performed with language group (bilingual, monolingual) as independent variable and four respective norm-references ATTA scores as dependent variable. The analyses revealed that bilinguals significantly outperformed monolinguals on the ATTA measure of flexibility [*F*(1,84) = 13.11, *p* < 0.05, η^2^ = 0.07]. No other significant performance differences were found. Considering significant differences between bilingual and monolingual samples in age and Russian iPNT scores (see above), the analysis was repeated with these variables as covariates. The ANCOVA revealed a significant group difference and an increased effect size for flexibility [*F*(1,82) = 11.32, *p* < 0.01, η^2^ = 0.12].

Kharkhurin (2009) argued that fluid intelligence might have a measurable contribution to the variation in creative performance of bilinguals and monolinguals. To exclude this option, ANOVA was performed with language group (bilingual, monolingual) as independent variable and Gf score as dependent variable. The analyses revealed no significant performance difference between bilingual and monolingual groups [*F*(1,84) = 0.94, *p* = 0.34, η^2^ = 0.01].

### Hypothesis II: TLP Effect Predicts Bilinguals’ Performance on Flexibility and Fluency

To test the second hypothesis, we determined how much of flexibility and fluency can be explained by the LMCA (measured by the TLP test) in bilingual and monolingual groups. Bilinguals demonstrated a significant correlation between the TLP effect score and flexibility (*r* = 0.38, *p* < 0.01), whereas monolinguals demonstrated no correlation between these measures (*r* = -0.01, *p* = 0.97). At the same time, bilinguals demonstrated marginally significant correlation between the TLP effect score and fluency (*r* = 0.22, *p* = 0.10), whereas monolinguals demonstrated no correlation between these measures (*r* = 0.06, *p* = 0.75). Therefore, hierarchical regression analyses were conducted to find the best fitting model for flexibility only. **Table 4** shows the betas and the model fit for each of the two regression analyses. In bilingual group, flexibility was negatively significantly predicted by response to CR pairs and positively significantly predicted by response to CU pairs. No significant effect was found in monolingual group.

**Table 4:** Beta’s and *F*’s for best-fitting model for flexibility as determined by a hierarchical regression of the predictor variables RT on critical related and critical unrelated pairs for bilingual and monolingual groups.

	Bilinguals		Monolinguals	
	β	*t*	β	*t*
RT on CR	–0.94	–3.00^∗^	–0.01	–0.03
RT on CU	0.92	2.93^∗^	–0.09	–0.22
	*F*(2,55) = 4.60, *p* < 0.05		*F*(2,25) = 0.12, *p* = 0.89	

*^∗^*p* < 0.01.*

This finding demonstrates that the TLP effect predicts bilinguals’ performance on flexibility. Note that the TLP score was calculated as the RT difference between responses to CU and CR pairs. Hence, increase in RT on CU pairs and decrease in RT on CR pairs signify increase in TLP score. In other words, slower response to CU pairs and faster response to CR pairs produce greater TLP effect.

### Hypothesis III: Bilinguals Demonstrate Greater TLP Effect than Monolinguals

To test the third hypothesis, ANCOVA was conducted with language group (bilingual, monolingual) as independent variable, TLP effect as dependent variable, and age and Russian iPNT scores as covariates. The analysis revealed no significant difference between language groups [*F*(1,82) = 0.30, *p* = 0.59, η^2^ = 0.004].

Moreover, no significant difference between RT to CR and CU pairs was found for either bilingual or monolingual groups.

### Hypothesis IV: TLP Effect Is Modulated by L2 Proficiency

In line with the forth hypothesis and considering that English was the language that was supposed to mediate the relatedness of the CR stimuli, high proficiency in this language was expected for the TLP effect to occur. A significant correlation between TLP effect score and iPNT score in English (*r* = 0.29, *p* < 0.05) in bilingual group supported this assumption. Note that the TLP effect score also significantly correlated with the age of acquisition of English (*r* = -0.30, *p* < 0.05).

Bilinguals were divided in high and moderate English proficiency groups using a median split on English iPNT scores (see second and third columns of **Table 3**). As **Table 3** indicates, they differed only in iPNT scores in English [*F*(1,56) = 80.59, *p* < 0.001, η^2^ = 0.59], age of acquisition of English [*F*(1,56) = 15.82, *p* < 0.001, η^2^ = 0.22], and TLP effect score [*F*(1,56) = 5.64, *p* < 0.05, η^2^ = 0.09]. Highly proficient bilinguals demonstrated significantly greater TLP effect than moderately proficient bilinguals did (Δ*M* = 15.07, *t* = 2.38, *p* < 0.05), but no significant difference with the monolinguals.

Moreover, although highly proficient bilinguals responded faster to the CR than to the CU pairs, this difference was insignificant (Δ*M* = -3.99, *t* = -0.94, *p* = 0.35). At the same time, moderately proficient bilinguals responded significantly slower to the CR than to the CU pairs (Δ*M* = 11.08, *t* = 2.34, *p* < 0.05).

## Discussion

The purpose of this study was to provide empirical evidence for the LMCA in bilinguals’ memory and to relate this cognitive mechanism to their divergent thinking. We obtained the following findings. First, bilinguals outperformed their monolingual counterparts on divergent thinking trait of flexibility. Second, bilinguals’ performance on this trait could be explained by the TLP effect. Third, bilinguals with high proficiency in English demonstrated greater TLP effect than did their counterparts with moderate English proficiency.

### Group Performance on Cognitive Flexibility

The LMCA model predicts that due to the lemma and/or lexical features mediated concept activation unrelated conceptual categories can be simultaneously engaged in problem solving. Simultaneous activation of multiple conceptual planes may prompt an individual to consider a variety of approaches to a problem concurrently; that is, it may promote cognitive flexibility. The finding of the present study demonstrating that bilinguals outperformed their monolingual counterparts on the ATTA’s flexibility supports this prediction.

It also contributes to a growing body of evidence cited in the beginning of this article, which reported bilinguals’ advantages on cognitive flexibility. For example, Adi-Japha et al. (2010) asked English–Hebrew and Arabic–Hebrew bilingual children and their Hebrew monolingual peers to draw a non-existent object (cf. Karmiloff-Smith, 1990). Bilinguals’ drawings exhibited a significantly higher rate of incorporating components from different categories into one drawing (i.e., interrepresentational flexibility) than monolinguals’ drawings.

### TLP Effects Explains Cognitive Flexibility

We found that the TLP effect predicts bilinguals’ performance on the ATTA’s flexibility. In bilingual memory, the TLP occurs between semantically unrelated words in one language if their translation equivalents are semantically related in the other language. Recall that the TLP test presented participants with pairs of Russian words. These pairs were constructed in such a way that the constituent words were not semantically related in Russian, but were semantically related in English. According to the LMCA model, Russian–English bilinguals should have processed these pairs as semantically related, whereas Russian monolinguals should have processed these words as semantically unrelated. Hence, bilinguals should have demonstrated greater TLP effect than monolinguals, which was at least partially found in the study (note that this effect was found only for those bilinguals who scored high on English proficiency test, which is addressed in the following section). Greater TLP effect signifies an ability to activate semantically unrelated words; that is, to access distant conceptual representations simultaneously. Instantaneous activation of multiple conceptual planes allows an individual to consider a variety of approaches to a problem concurrently; that is, it facilitates cognitive flexibility.

At the same time, if the LMCA facilitates simultaneous activation of unrelated conceptual representations, bilingual participants’ TLP performance was expected to relate not only to flexibility, but also to fluency in divergent thinking. Recall that fluency measures the ability to produce quantities of ideas. Simultaneous activation of unrelated concepts therefore, should trigger production of a large number of solutions to a problem. However, we did not find that the TLP effect predicts fluency in divergent thinking. This finding sheds some light on the nature of the LMCA.

This mechanism is supposed to spread activation between distant conceptual categories such as [crime] and [animal] in the example above. The finding that the TLP predicts cognitive flexibility suggests that these categories can be accessed. However, as the activation flow travels between lexical and conceptual representations, it may become weaker. When weak activation reaches distant conceptual categories, it may be not sufficient to generate and retrieve solutions to divergent thinking problem. The LMCA provides access to distant conceptual categories and thereby promotes cognitive flexibility. However, this mechanism does not provide enough activation for individual conceptual representations to stimulate fluency. Recall from the discussion of the conceptual memory above that only those conceptual features that receive enough activation are selected for conscious processing. This would explain why bilinguals in the present study demonstrated only marginally significant correlation between the TLP and fluency.

### Factors in Bilingual Development Influencing the LMCA

The activation flow in bilingual memory can be boosted by stronger connections between conceptual and lexical systems. These connections can be strengthen in the course of bilingual development. The findings of a significant correlation between the TLP effect and proficiency in English and age of acquisition of English suggest at least two developmental factors facilitating the LMCA.

#### Age of L2 Acquisition

A negative correlation between the age of L2 acquisition and the TLP effect suggests that this factor may have an impact on the structure of bilingual memory. Kharkhurin (2007) explained this as following:

“Individuals who acquired both of their languages early in life may develop a greater sensitivity to underlying concepts and more refined connections between lexical and conceptual representations. If bilinguals acquired both of their languages early and underwent an equal development in both languages, they might be able to establish equally strong direct links from both lexicons to the conceptual system. These links can be reinforced by a constant exposure to both languages in combination with frequent language switching. Thus, bilinguals who acquired their languages early in life would have two equally developed lexical systems connected to a shared conceptual one. This presumably fosters the <LMCA> by providing fast routing of information exchange between both lexicons and the concepts.

On the other hand, individuals who acquired their L2 later in life, first establish the links between their L1 lexicon and their conceptual system. During L2 learning they initially access the meanings for L2 words through L1 and only later become able to conceptually mediate L2 directly. The shift from reliance on L1 to direct conceptual processing of L2 may result in creating an asymmetry in lexical access (see Kroll and de Groot, 1997, for detailed discussion). The late bilinguals would have more lexical-conceptual connections from L1 than from L2, and the strength of these links would be different for first and second languages. Due to lexical access asymmetry, more conceptual features can be accessed through L1 than through L2. Since the vast majority of the conceptual system in late bilinguals was established during L1 acquisition, and since L2 lexical features were mapped to the conceptual features through the L1 lexical-conceptual route, there might be fewer shared conceptual features that have direct links from both lexicons in the memory of individuals who acquired L2 later in life. This may result in a less efficient <LMCA>, and consequently in a poorer divergent thinking performance” (pp. 201–202).

Indeed, the studies show that bilinguals who acquired L2 earlier in life outperformed their counterparts who acquired L2 later on various divergent thinking measures. A group of Armenian–Russian bilinguals who learned both languages simultaneously scored higher on flexibility and originality than their counterparts who started to learn one of the two languages 2–4 years later (Kostandyan and Ledovaya, 2013). There is also evidence that Russian–English bilinguals who acquired L2 at a younger age scored higher on fluency and flexibility (Kharkhurin, 2008). Similarly, bilinguals who acquired their L2 by the age of six tended to solve insight problems more readily than their counterparts who acquired L2 after this age (Cushen and Wiley, 2011).

#### Language Proficiency

The finding of a positive correlation between the proficiency in L2 and the TLP effect suggests that this factor may have an impact on the functioning of bilingual memory. Kharkhurin (2007) explained this as following:

“The degree of linguistic skills may influence the intensity of the lexical access: greater language proficiency may result in establishing stronger and more elaborate links to the conceptual system. As a result, more concepts become readily available for <LMCA>. Following this assumption, bilinguals who attained a high expertise in both languages would have stronger and more efficient links between lexical and conceptual levels then those who were not able to develop any of their languages to a high degree” (p. 202). This assumption is supported by a significantly higher TLP performance by bilingual participants who were highly proficient in English in comparison with their counterparts who were moderately proficient in this language.

Kharkhurin (2007) continues: “Thus, bilinguals highly proficient in both languages would employ the <LMCA> mechanism more effectively and therefore may show greater divergent thinking performance compared with their less proficient counterparts” (p. 202). The studies support this idea by showing that bilinguals with high proficiency in both languages outperform their less proficient counterparts on divergent thinking measures. Bilinguals highly proficient in both English and Russian performed better on elaboration than their less proficient peers (Kharkhurin, 2008). Similarly, Farsi–English bilinguals highly proficient in both languages outperformed their unbalanced and moderately proficient peers on fluency (Kharkhurin, 2009). Lee and Kim (2011) found that more balanced Korean–English bilinguals obtained higher creativity scores than their less balanced counterparts. Kharkhurin (2011) compared bilinguals with different levels of proficiency in English. The study demonstrated that bilinguals with greater linguistic skills in English tended to score higher on originality and revealed more unstructured imagination (cf. Ward, 1994). All these findings fit with the threshold hypothesis (Cummins, 1976) postulating that bilinguals need to achieve a minimum (age-appropriate) proficiency threshold in both of their languages to reveal cognitive advantages.

Altogether, both the age of language acquisition and language proficiency seem to have an impact on the structure and functioning of bilingual memory. The age of L2 acquisition may stipulate the directions of lexical-conceptual routes. The proficiency in L1 and L2 may stipulate the strength of the connections between the conceptual and lexical systems. As a result, more conceptual representations become readily available for the LMCA, which in turn may promote cognitive flexibility.

### Construct Validity of the TLP Test

The TLP test involves a complex procedure, which is based on several theoretical assumptions. Therefore, it is important to discuss the construct validity of this test. It was hypothesized that if a special structure of bilingual memory encourages the LMCA, bilinguals should demonstrate greater TLP effect than their monolingual peers. However, no effect was found either in bilingual or in monolingual group, and no group difference was found either.

Then, it was supposed that this effect could be masked by bilinguals’ different levels of proficiency in English, which was the mediating language in the TLP test. Indeed, bilinguals with higher level of English proficiency demonstrated relatively greater TLP effect than did their peers with moderate proficiency in English, but there was no significant difference in their reaction to CR and CU pairs; that is, even the highly proficient bilinguals demonstrated no TLP effect. At the same time, faster responses to CR pairs and slower responses on CU pairs predicted cognitive flexibility in bilingual, but not in monolingual group.

A possible explanation of this discrepancy refers to the language organization in the memory of bilingual individuals in the present sample. Recall that the TLP procedure assumed the activation flow from the prime in L1 to its translation equivalent in L2 to translation equivalent of a target in L2 to the target in L1 (see **Figure 3**). Therefore, the TLP effect may occur only when connections between lexical and conceptual systems in both languages are strong and efficient. At the same time, participants in our sample were sequential^3^ bilinguals who acquired English primarily in a classroom setting (i.e., decontextualized environment; cf. Pavlenko, 2000), and were less proficient in English than in Russian. According to the discussion in the previous subsection, both the age of acquisition and language proficiency may influence the connections between lexical and conceptual representations in bilingual memory. Therefore, bilinguals in the present sample could have asymmetrical lexical access, which interrupted the activation flow between lexical and conceptual systems. To test this hypothesis, the TLP procedure needs to be tested with a sample of simultaneous bilinguals or at least those who acquired their L2 in environment where they used this language in everyday life.

## Conclusion

This study was the first attempt of an empirical investigation of the LMCA in bilingual memory as a cognitive mechanism facilitating divergent thinking. The findings propose that due to the lemma mediated concept activation unrelated conceptual categories can be simultaneously engaged in creative problem solving, which eventually may promote cognitive flexibility. Two factors in cross-linguistic experience (age of language acquisition and language proficiency) could have an impact on the connections between lexical and conceptual representations in bilingual memory. The study also introduced a procedure that tested the TLP effect in bilingual memory. Previously mentioned cross-language studies use translation (review in Altarriba and Basnight-Brown, 2007) and semantic (review in Kroll and Tokowicz, 2005) priming paradigms, which assessed relatedness between words in bilinguals’ different languages. The TLP test introduces a paradigm aiming at assessing relatedness between words in the same language through bilinguals’ other language. That is, in addition to L1–L2 link it tests L1–L2–L1 link in bilingual memory. However, the study questioned construct validity of the TLP paradigm and recommended additional testing.

The findings of the present study lay another brick into multilingual creative cognition paradigm (Kharkhurin, 2015c). According to this framework, acquisition and use of multiple languages has an impact on the structure and/or functioning of an individual’s memory. Strengthening of certain cognitive functions may have impact on creativity fostering traits such as cognitive flexibility, tolerance for ambiguity, open-mindedness, and intrinsic motivation (see Kharkhurin, 2016b, for a discussion). Hence, multilingual practice may facilitate creative potential.

This conclusion can have important ramifications in a context of a widely discussed topic in both multilingualism and creativity research that comes from pedagogical considerations. Kharkhurin (2015a) explains: “It is evident that the creativity-fostering programs operate separately from those offering bilingual instruction, and researchers and teachers have mutually exclusive training. They are educated in either creativity or language related disciplines. <Overall,> the academic community generally disregards the potential relationship between bilingualism and creativity. Similarly, the benefits of merging programs fostering creative potential and bilingual abilities seem to escape the attention of the educators. However, the efficacy of a program combining both efforts can be directly inferred from the research <in multilingual creative cognition>. <…> Therefore, by combining bilingual and creative education <strategies>, a far greater synergy could be generated – a bilingual creative education program would capitalize on the assets of both forms of education to establish an effective and comprehensive curriculum” (pp. 46–47; see discussion of this program in Kharkhurin, 2012a, 2015b, 2016a).

## Author Contributions

The manuscript was entirely prepared by the author.

## Conflict of Interest Statement

The author declares that the research was conducted in the absence of any commercial or financial relationships that could be construed as a potential conflict of interest.

## Figures and Tables

**APPENDIX A:**
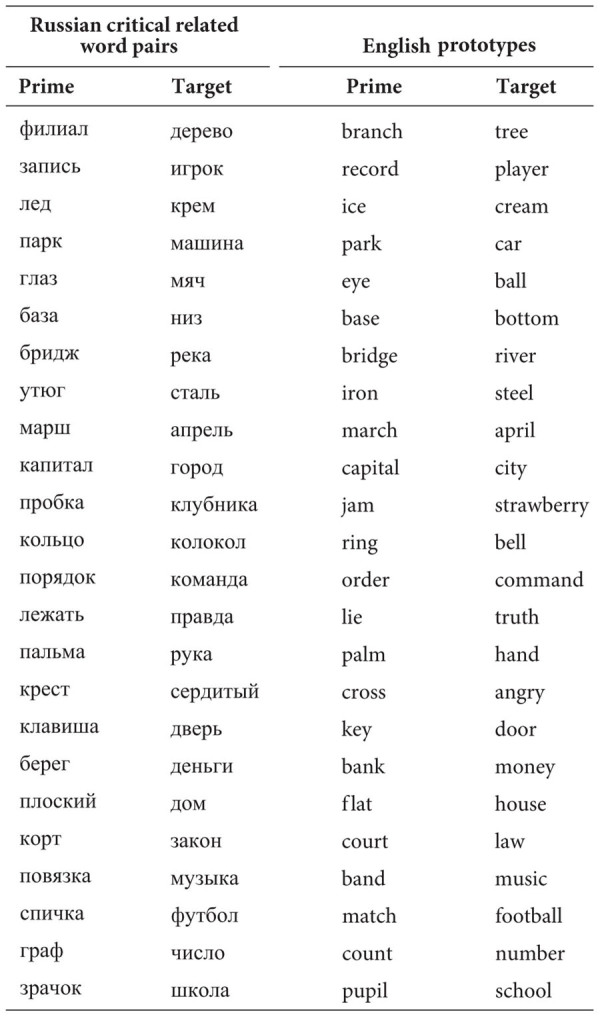
**Russian critical related word pairs and their English prototypes**.

## Footnotes

1 Kharkhurin (2014) challenged this definition as being biased by a Western perception of creativity. He proposed an alternative four-criterion construct of creativity, which in addition to novelty and utility considers two other characteristics typical for Eastern perception of creativity: aesthetics and authenticity.

2 The original English version can be found online by following this link: http://surveys.harhur.com/index.php?r=survey/index/sid/782753&lang=en

3 Traditionally, the distinction is made between simultaneous and sequential bilinguals (McLaughlin, 1984). The simultaneous bilinguals learn both of their languages from the onset of language acquisition. The sequential bilinguals learn their L2 after age of 5, when the basic components of L1 are already in place. The sequential bilinguals are further divided into early and late ones, reflecting the age at which L2 acquisition occurred (Genesee, 1978).
